# SLC25A22 as a Key Mitochondrial Transporter Against Ferroptosis by Producing Glutathione and Monounsaturated Fatty Acids

**DOI:** 10.1089/ars.2022.0203

**Published:** 2023-07-17

**Authors:** Yang Liu, Yuan Wang, Zhi Lin, Rui Kang, Daolin Tang, Jiao Liu

**Affiliations:** ^1^ The DAMP Laboratory, Third Affiliated Hospital of Guangzhou Medical University, Guangzhou, China.; ^2^ Guangzhou Municipal and Guangdong Provincial Key Laboratory of Protein Modification and Degradation, Guangzhou Medical University, Guangzhou, China.; ^3^ Department of Pediatrics, The Third Xiangya Hospital, Central South University, Changsha, China.; ^4^ Department of Surgery, UT Southwestern Medical Center, Dallas, Texas, USA.

**Keywords:** SLC25A22, mitochondria, ferroptosis, lipid peroxidation

## Abstract

**Aims:**

Ferroptosis, a type of oxidative cell death driven by unlimited lipid peroxidation, is emerging as a target for cancer therapy. Although mitochondrial dysfunction may lead to ferroptosis, the underlying molecular mechanisms and metabolic pathways for ferroptosis are incompletely understood. Here, we identify solute carrier family 25 member 22 (SLC25A22), a mitochondrial glutamate transporter, as a driver of ferroptosis resistance in pancreatic ductal adenocarcinoma (PDAC) cells.

**Results:**

The downregulation of SLC25A22 expression was associated with increased sensitivity to ferroptosis, but not to apoptosis. Mechanistically, on the one hand, SLC25A22-dependent NAPDH synthesis blocks ferroptotic cell death in PDAC cells through mediating the production of glutathione (GSH), the most important hydrophilic antioxidant. On the other hand, SLC25A22 promotes the expression of stearoyl-CoA desaturase in PDAC cells in an AMP-activated protein kinase-dependent manner, resulting in the production of antiferroptotic monounsaturated fatty acids (MUFAs). The animal study further confirms that SLC25A22 inhibits ferroptosis-mediated tumor suppression.

**Innovation:**

SLC25A22 is a novel metabolic repressor of ferroptosis by producing GSH and MUFAs.

**Conclusion:**

These findings establish a previously unrecognized metabolic defense pathway to limit ferroptotic cell death *in vitro* and *in vivo*. *Antioxid. Redox Signal.* 39, 166–185.

## Introduction

Pancreatic cancer remains a clinical challenge due to its late diagnosis, limited treatment options, and poor prognosis (Bear et al., 2020). The predominant pathological type of pancreatic cancer is pancreatic ductal adenocarcinoma (PDAC), which is driven by multiple genetic mutations, particularly in the oncogene *KRAS* (Li et al., 2021b; Mizrahi et al., 2020). For patients with advanced PDAC, chemotherapy alone or in combination with radiation or immunotherapy is the primary treatment option (Neoptolemos et al., 2018). However, the clinical benefit of these treatments is limited, and the current 5-year survival rate for PDAC patients is ∼10% (Siegel et al., 2022).

A potential cause of chemical refractory outcomes is the emergence of multidrug resistance, especially resistance to apoptotic cell death (Yang et al., 2021). Therefore, developing drugs or methods to induce nonapoptotic cell death may be an effective way to kill PDAC cells and overcome drug resistance (Chen et al., 2021e).

With the development of biotechnology and experimental methods, an increasing number of nonapoptotic cell death types (*e.g.,* necroptosis, ferroptosis, pyroptosis, alkaliptosis, and cuproptosis) have been identified (Tang et al., 2019). Among them, pharmacologically induced ferroptosis is increasingly recognized as a way to kill multidrug-resistant cancer cells (Chen et al., 2021b). Unlike caspase-dependent apoptosis, ferroptosis is an iron-dependent oxidative cell death caused by lipid peroxidation, which is controlled by an integrated oxidative and antioxidant system (Liu et al., 2021a).

Ferroptosis is regulated by multiple subcellular organelles (Chen et al., 2021c), and mitochondria are thought to play a complex role in shaping the metabolic response to ferroptosis (Gao et al., 2019). In addition to energy metabolism, mitochondria control the production of iron, amino acids, fatty acids, and antioxidants, leading to changes in ferroptosis sensitivity (Li et al., 2021a; Song et al., 2021; Yuan et al., 2016a). The identification and characterization of key mitochondrial proteins that regulate ferroptosis sensitivity are critical for the development of targeted therapy.

The mitochondrial solute carrier 25 (SLC25) family consists of 53 members that transport diverse small molecules (*e.g.,* metabolites, nucleotides, and cofactors) between mitochondria and the cytoplasm (Ruprecht and Kunji, 2020). Aberrant expression of the SLC25 family is associated with mitochondrial stress, leading to cell survival or death (Kunji et al., 2020). Dysfunction in the SLC25 family is implicated in a variety of human diseases and pathological conditions, including PDAC (Rochette et al., 2020).

Previous studies have suggested that the solute carrier family 25 member 10 (SLC25A10, also known as DIC) and solute carrier family 25 member 11 (SLC25A11, also known as OGC) transport glutathione (GSH) into mitochondria, and inhibiting their function can enhance ferroptosis (Jang et al., 2021; Ta et al., 2022). In addition, SLC25A28 is an iron transporter in the inner mitochondrial membrane, and blocking its activity prevents erastin-induced ferroptosis in hepatic stellate cells (Zhang et al., 2020). Despite these findings, the specific role of SLC25 family members in ferroptosis in PDAC remains unclear.

Here, we report that SLC25 family member 22 (SLC25A22) plays an essential role in blocking ferroptosis, mainly through maintaining GSH production in human PDAC cells *in vitro* and *in vivo*. We also demonstrate functional significance of the axis of adenosine triphosphate (ATP)–AMP-activated protein kinase (AMPK)–stearoyl-CoA desaturase (SCD, also known as SCD1) in mediating ferroptosis resistance in human PDACs. Consequently, genetic deletion of SLC25A22 promotes ferroptosis sensitivity *in vitro* and in xenograft mouse models. These findings establish a novel mitochondrial metabolic pathway to control ferroptosis sensitivity in PDAC.

## Results

### The downregulation of SLC25A22 during ferroptosis

**FIG. 1.**
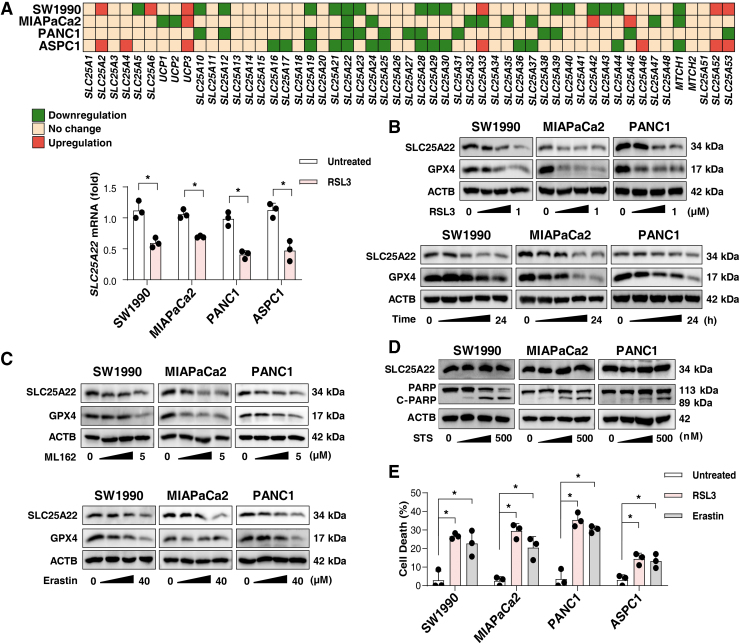
**The selective downregulation of SLC25A22 during ferroptosis. (A)** The indicated PDAC cells were treated with RSL3 (1 μ*M*) or vehicle for 24 h, and the mRNA of the SLC25A family was analyzed (*top*). Downregulation: 2^−ΔΔCt^ <0.7. No change: 0.7 ≤ 2^−ΔΔCt^ <1.5. Upregulation: 2^−ΔΔCt^ ≥1.5. Analysis of SLC25A22 expression in indicated PDAC cells after treatment with RSL3 (1 μ*M*) for 24 h (*bottom*). **(B)** Analysis of SLC25A22 and GPX4 protein levels in indicated PDAC cells after treatment with RSL3 (0.25, 0.5, and 1 μ*M*) for 24 h (*top*) or after treatment with RSL3 (1 μ*M*) for different times (3, 6, 12, and 24 h; *bottom*). **(C)** Analysis of SLC25A22 and GPX4 protein levels in PDAC cells after treatment with erastin (10, 20, and 40 μ*M*; *top*) or ML162 (1.25, 2.5, and 5 μ*M*; *bottom*) for 24 h. **(D)** Analysis of indicated protein expression in PDAC cells after treatment with staurosporine (125, 250, and 500 n*M*) for 24 h. **(E)** Cell death of indicated PDAC cells after treatment with RSL3 (1 μ*M*) or erastin (20 μ*M*) for 24 h. Data in **(A, E)** are presented as the mean ± SD of three technical replicates from one representative dataset of three independent experiments. Statistical significance was analyzed using Student's *t*-test **(A)**, or one-way ANOVA with Dunnett's *post hoc* test **(E)**. *p < 0.05. ANOVA, analysis of variance; GPX4, glutathione peroxidase 4; PDAC, pancreatic ductal adenocarcinoma; SD, standard deviation; SLC25A22, solute carrier family 25 member 22.

To understand the roles of the SLC25 family in ferroptosis, we quantified the expression of 53 members of the SLC25 family in 4 human PDAC cell lines (ASPC1, MIAPaCa2, PANC1, and SW1990) with the classical ferroptosis agonist RSL3. A quantitative real-time polymerase chain reaction (qPCR) analysis showed that the mRNA level of SLC25A22 (but not the other 52 genes of the SLC25 family) was significantly reduced (2^−ΔΔCt^ <0.7) in these tested PDAC cells after RSL3 treatment (Fig. 1A).

Western blots confirmed that RSL3 dose- and time dependently inhibited the expression of SLC25A22 protein in MIAPaCa2, PANC1, and SW1990 cells (Fig. 1B). However, the downregulation of SLC25A22 protein was not observed in RSL3-treated ASPC1 cells (Supplementary Fig. S1A), indicating that the expression of SLC25A22 protein in response to RSL3 may be affected in a transcription-independent manner. Consistent with previous studies (Li et al., 2021c), glutathione peroxidase 4 (GPX4) protein expression was also inhibited in PDAC cells by RSL3 (Fig. 1B).

Similar trends in SLC25A22 protein expression inhibition were observed in PDAC cells after treatment with other ferroptosis inducers, such as erastin and ML162 (Fig. 1C). However, the apoptosis inducer (staurosporine [STS]) had no effect on SLC25A22 expression (Fig. 1D), supporting that SLC25A22 downregulation is a marker of ferroptosis. As a positive control, STS induced cleavage of poly(ADP-ribose) polymerase 1 (PARP1) protein (Fig. 1D).

Moreover, we observed that the cell lines with downregulated SLC25A22 protein (MIAPaCa2, PANC1, and SW1990) were more susceptible to cell death induced by RSL3 or erastin than ASPC1 cells (Fig. 1E). Among the four cell lines we tested, PANC1 displayed the highest sensitivity to ferroptosis (Fig. 1E). This suggests that targeted downregulation of the SLC25A22 protein may selectively promote ferroptosis.

### SLC25A22 is a repressor of ferroptosis

**FIG. 2.**
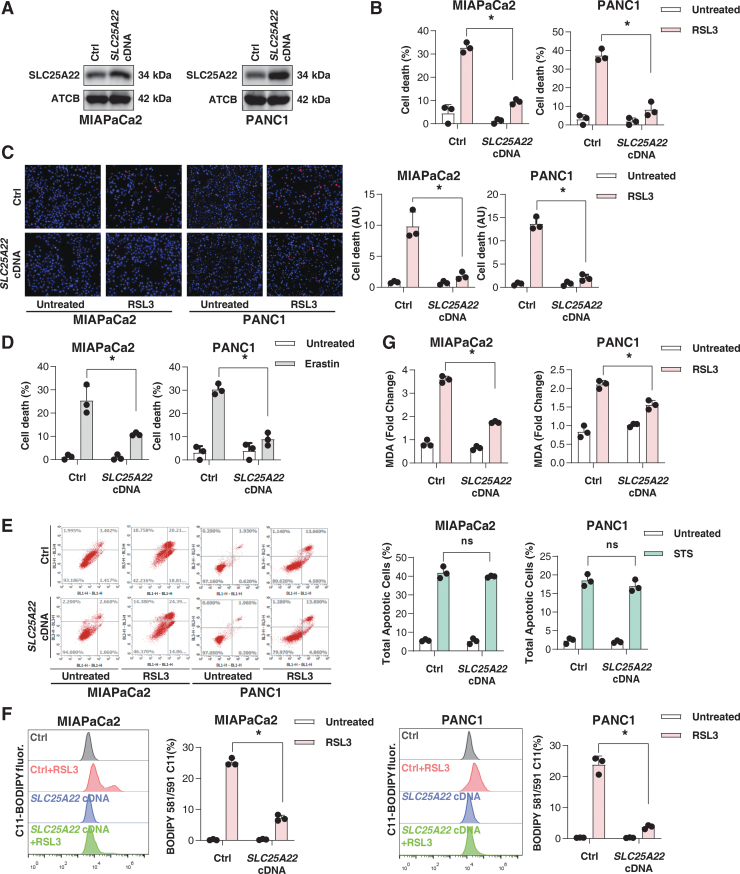
**The overexpression of SLC25A22 inhibits ferroptosis in PDAC cells. (A)** Analysis of SLC25A22 protein expression in indicated PDAC cells. **(B)** Cell viability of indicated PDAC cells after treatment with RSL3 (1 μ*M*) for 24 h. **(C)** Measuring cell death by PI staining in indicated PDAC cells after treatment with RSL3 (1 μ*M*) for 24 h. The *panel* shows representative images of PI staining in PDAC cells. **(D)** Cell viability of indicated PDAC cells after treatment with erastin (20 μ*M*) for 24 h. **(E)** Apoptosis assay using the fluorescent probes annexin V-FITC and PI in indicated PDAC cells after treatment with staurosporine (200 n*M*) for 24 h. The *chart* to the *right* shows the total apoptosis levels in PDAC cells after staurosporine (200 n*M*, 24 h) treatment. **(F, G)** The indicated PDAC cells were treated with RSL3 (1 μ*M*) for 24 h, and then intracellular lipid ROS **(F)** and MDA **(G)** were measured. Data in **(B–G)** are presented as the mean ± SD of three technical replicates from one representative dataset of three independent experiments. Statistical significance was analyzed using two-way ANOVA with Dunnett's *post hoc* test. *p < 0.05. MDA, malondialdehyde; PI, propidium iodide.

We chose to focus our investigation on MIAPaCa2 and PANC1 cells, as they exhibit greater tumor aggressiveness than other human PDAC cell lines (Deer et al., 2010). To determine the effect of SLC25A22 on ferroptosis, we ectopically expressed SLC25A22 in MIAPaCa2 and PANC1 cells (Fig. 2A). The overexpression of SLC25A22 inhibited RSL3-induced cell death in MIAPaCa2 and PANC1 cells (Fig. 2B, C). Similarly, the upregulation of SLC25A22 inhibited erastin-induced cell death in these PDAC cells (Fig. 2D). In contrast, the overexpression of SLC25A22 failed to affect STS-induced apoptosis as shown by flow cytometry assay (Fig. 2E).

To gain insight into SLC25A22-mediated resistance to RSL3, we assayed lipid peroxidation using two methods. As with flow cytometry assays using the fluorescent probe C11-BODIPY, the quantification of the lipid oxidation end product malondialdehyde (MDA) also revealed that RSL3 induced lipid peroxidation in wild-type PDAC cells (Fig. 2F, G). This process was significantly blocked in SLC25A22-overexpressed MIAPaCa2 and PANC1 cells (Fig. 2F, G). Therefore, SLC25A22 can inhibit lipid peroxidation during ferroptosis.

**FIG. 3.**
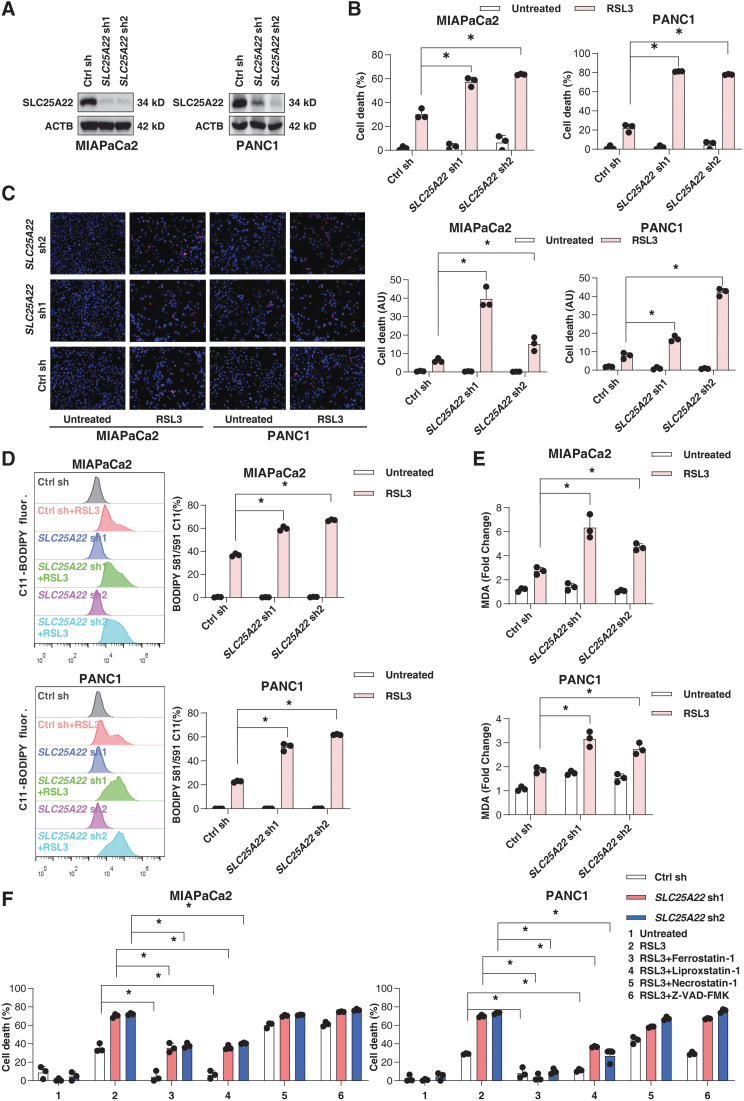
**The knockdown of SLC25A22 promotes ferroptosis in PDAC cells. (A)** Analysis of SLC25A22 protein expression in indicated PDAC cells. **(B)** Cell viability of indicated PDAC cells after treatment with RSL3 (1 μ*M*) for 24 h. **(C)** Measuring cell death by PI staining in indicated PDAC cells after treatment with RSL3 (1 μ*M*) for 24 h. The *panel* shows representative images of PI staining in PDAC cells. **(D, E)** The indicated PDAC cells were treated with RSL3 (1 μ*M*) for 24 h, and then intracellular lipid ROS **(D)** and MDA **(E)** were measured. **(F)** Cell viability of indicated PDAC cells after treatment with RSL3 (1 μ*M*) in the absence or presence of ferrostatin-1 (1 μ*M*), liproxstatin-1 (500 n*M*), necrostatin-1 (10 μ*M*), and Z-VAD-FMK (10 μ*M*) for 24 h. Data in **(B–F)** are presented as the mean ± SD of three technical replicates from one representative dataset of three independent experiments. Statistical significance was analyzed using two-way ANOVA with Dunnett's *post hoc* test. *p < 0.05.

To further test the hypothesis of SLC25A22 as a ferroptosis suppressor, we used specific lentiviral shRNA to inhibit the expression of SLC25A22 in MIAPaCa2 and PANC1 cells (Fig. 3A). Compared with a control group, SLC25A22 depletion increased RSL3-induced cell death (Fig. 3B, C) and lipid peroxidation (Fig. 3D, E). The increased ferroptosis sensitivity in SLC25A22-knockdown PDAC cells was reversed by ferroptosis inhibitors (liproxstatin-1 and ferrostatin-1), rather than by an apoptosis inhibitor (Z-VAD-FMK) or necroptosis inhibitor (necrostatin-1; Fig. 3F). These data suggest that the increased sensitivity to cell death in SLC25A22-knockdown cells is caused by ferroptosis.

**FIG. 4.**
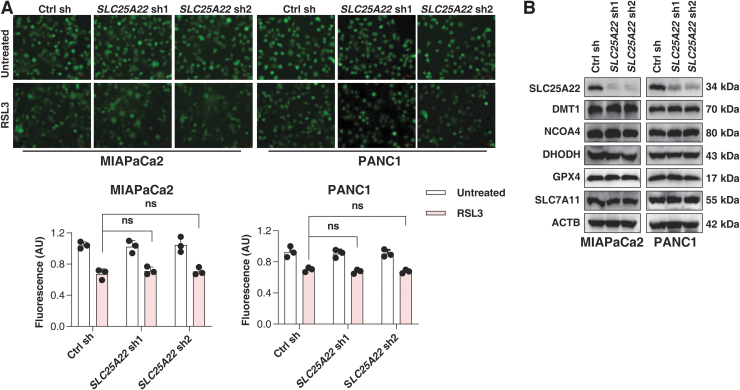
**SLC25A22 inhibits ferroptosis independently of iron. (A)** The indicated PDAC cells were treated with RSL3 (1 μ*M*) for 24 h, and the intracellular iron was measured. The *panel* shows representative images of PGSK staining in PDAC cells. **(B)** Analysis of protein levels in indicated PDAC cells. Data in **(A)** are presented as the mean ± SD of three technical replicates from one representative dataset of three independent experiments. Statistical significance was analyzed using two-way ANOVA with Dunnett's *post hoc* test. ns, not significant.

Since iron accumulation contributes to ROS production and subsequent lipid peroxidation during ferroptosis (Dixon et al., 2012), we also determined the intracellular Fe^2+^ content in PDAC cells (Fig. 4A). The depletion of SLC25A22 had no significant effect on RSL3-induced intracellular Fe^2+^ accumulation (Fig. 4A). Iron metabolism-associated proteins, such as SLC11A2 (also known as DMT1) and nuclear receptor coactivator 4 (NCOA4), were also not significantly altered by silencing SLC25A22 (Fig. 4B). These findings suggest that SLC25A22 does not inhibit ferroptosis by regulating iron accumulation.

### SLC25A22 mediates antioxidant production during ferroptosis

**FIG. 5.**
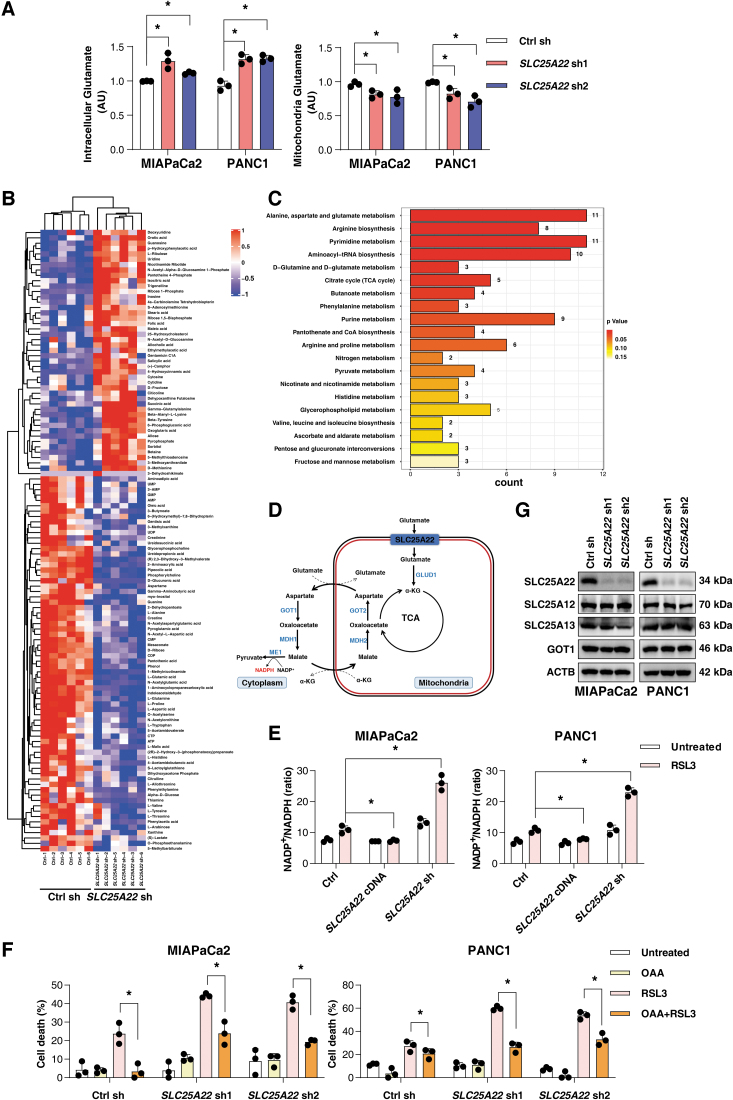
**SLC25A22 mediates antioxidant production during ferroptosis. (A)** The cytosolic (*left*) and mitochondrial (*right*) glutamate levels were measured in indicated PDAC cells. **(B)** Heatmap of the differential metabolite levels in SLC25A22-knockdown PANC1 cells. **(C)** KEGG analysis shows the top 20 different metabolic pathways. **(D)** The scheme depicting how glutamate forms NADPH through the TCA cycle and malate–aspartate shuttle. **(E)** The indicated PDAC cells were treated with RSL3 (1 μ*M*) for 24 h, and then the intracellular NADP^+^/NADPH ratio was assayed. **(F)** Cell viability of indicated PDAC cells after treatment with RSL3 (1 μ*M*) in the absence or presence of oxaloacetate (1 m*M*) for 24 h. **(G)** Analysis of protein levels in indicated PDAC cells. Data in **(A, E–F)** are presented as the mean ± SD of three technical replicates from one representative dataset of three independent experiments. Statistical significance was analyzed using two-way ANOVA with Dunnett's *post hoc* test. *p < 0.05. KEGG, Kyoto Encyclopedia of Genes and Genomes; TCA, tricarboxylic acid.

The primary function of SLC25A22 is to catalyze the transport of glutamate associated with a proton (H^+^) through the inner mitochondrial membrane (Fiermonte et al., 2002). As expected, the levels of mitochondrial glutamate were decreased, whereas the intracellular glutamate levels were increased in SLC25A22-knockdown MIAPaCa2 and PANC1 cells (Fig. 5A).

Because SLC25A22 functions as a transporter that catalyzes the uptake of glutamate across the inner mitochondrial membrane, we reasoned that SLC25A22-mediated glutamate import may play a role in glutamate metabolism in the mitochondria. To explore the effect of SCL25A22 on metabolism, we performed a global metabolomics analysis to detect the changes after the depletion of SLC25A22. The heatmap showed that 116 metabolites were changed (Fig. 5B). Pathway analysis further showed that with the depletion of SLC25A22 in PANC1 cells, the pathways of alanine, aspartate, and glutamate were significantly altered (Fig. 5C). These findings suggest that SLC25A22 may play an important role in malate–aspartate shuttling, mediating the production of aspartate (Fig. 5D).

A recent study showed that the knockdown of SLC25A22 suppressed aspartate synthesis and reduced NADPH production *via* the tricarboxylic acid (TCA) cycle in colorectal cancer cells (Wong et al., 2016). Thus, we hypothesized that SLC25A22 inhibits ferroptosis through mediating the product of NADPH. Indeed, the depletion of SLC25A22 increased the NADP^+^/NADPH ratio, which was further increased after RSL3 treatment in SLC25A22-knockdown MIAPaCa2 and PANC1 cells (Fig. 5E).

In contrast, the upregulation of SLC25A22 inhibited the depletion of NADPH after the treatment with RSL3 (Fig. 5E). We also found that the supplementation of culture media with oxaloacetate partly inhibited cell death in SLC25A22-knockdown cells during ferroptosis (Fig. 5F). Aspartate-derived cytosolic oxaloacetate participated in the malate–aspartate shuttle, which promoted the production of NADPH in PDAC cells (Fig. 5D). However, the depletion of SLC25A22 did not affect the expression of SLC25A12, SLC25A13, and glutamic-oxaloacetic transaminase 1 (Fig. 5G). Therefore, SLC25A22 does not directly regulate the function of malate-aspartate shuttle–related genes to affect NADPH production.

As the pentose phosphate pathway (PPP) has been proposed as a potential source of NADPH (Paul et al., 2022), we investigated the expression of glucose-6-phosphate dehydrogenase (G6PD), which is the enzyme responsible for the first step of the PPP to inhibit ferroptosis (Cao et al., 2021). Our results show that SLC25A22 upregulates the expression of G6PD in PANC1 and MIAPaCa2 cells (Supplementary Fig. S1B), suggesting a potential role for G6PD-mediated PPP in the antiferroptotic effects mediated by SLC25A22.

**FIG. 6.**
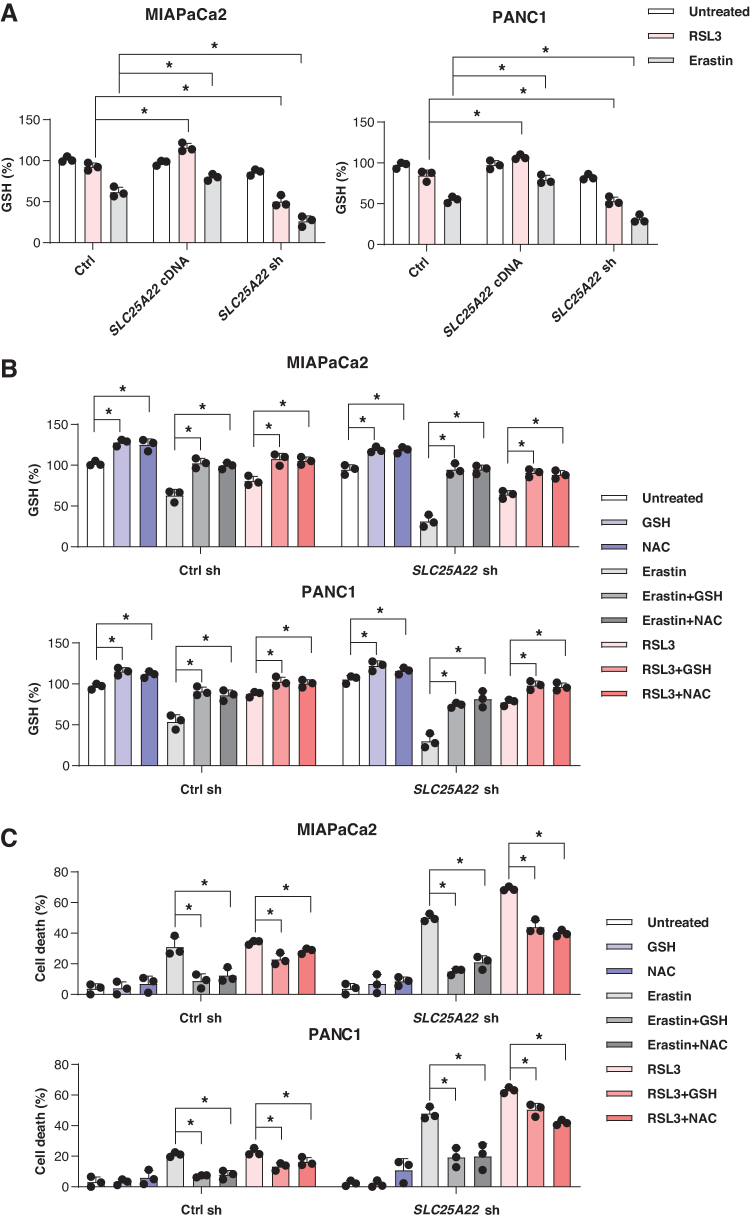
**SLC25A22 promotes the production of GSH. (A)** The indicated PDAC cells were treated with RSL3 (1 μ*M*) or erastin (20 μ*M*) for 24 h, and then cell GSH levels were assayed. **(B, C)** The GSH levels and the cell viability of indicated PDAC cells after treatment with RSL3 (1 μ*M*) or erastin (20 μ*M*) in the absence or presence of GSH (1 m*M*) and NAC (1 m*M*) for 24 h. Data in **(A–C)** are presented as the mean ± SD of three technical replicates from one representative dataset of three independent experiments. Statistical significance was analyzed using two-way ANOVA with Dunnett's *post hoc* test. *p < 0.05. GSH, glutathione; NAC, *N*-acetylcysteine.

NADPH is used for the reduction of GSSG to GSH by glutathione-disulfide reductase (Fig. 5D) (Koppula et al., 2021). As expected, RSL3- or erastin-induced GSH depletion was increased in SLC25A22-knockdown MIAPaCa2 and PANC1 cells (Fig. 6A). In contrast, the overexpression of SLC25A22 inhibited RSL3- or erastin-induced GSH depletion (Fig. 6A). Compared with the control group, the level of GSH was increased in SLC25A22-overexpressed PDAC cells after the treatment with RSL3 (Fig. 6A).

To investigate the pathway by which SLC25A22 mediates GSH synthesis involved in ferroptosis, we examined the effects of *N*-acetylcysteine (NAC) amide (a precursor of GSH) and GSH on RSL3- or erastin-induced growth inhibition in SLC25A22-knockdown PDAC cells. NAC and GSH significantly rescued erastin-induced GSH depletion and ferroptotic cell death, and partially rescued RSL3 resistance in SLC25A22-knockdown MIAPaCa2 and PANC1 cells (Fig. 6B, C). Although NAC may have GSH-independent functions (Ezerina et al., 2018), our findings support that SLC25A22 is a mitochondrial repressor of ferroptosis by producing the antioxidants NADPH and GSH.

RSL3 is a direct inhibitor of GPX4, but not system xc^−^ (Yang et al., 2014). Western blot analysis revealed that the expression of GPX4 and SLC7A11 was not changed by the depletion of SLC25A22 (Fig. 4B). The depletion of SLC25A22 had no effects on the expression of dihydroorotate dehydrogenase (DHODH) (quinone; Fig. 4B), a mitochondrial repressor of ferroptosis by reducing ubiquinone to ubiquinol (Mao et al., 2021). The overexpression of SLC25A22 also failed to restore ferroptosis resistance in DHODH-silenced cells (Supplementary Fig. S1C). Thus, SLC25A22 plays a role in mediating GSH synthesis in PDAC cells without affecting the protein expression of GPX4, SLC7A11, and DHODH.

### SLC25A22 upregulates the expression of SCD

Lipid peroxidation, a hallmark of ferroptosis, is caused by a complex process of lipid metabolism (Chen et al., 2021d; Lin et al., 2021). Previous studies have revealed that polyunsaturated fatty acids (PUFAs), synthesized by acyl-CoA synthase long-chain family member 4 (ACSL4), are the preferred substrates for lipid peroxidation during ferroptosis (Doll et al., 2017; Yuan et al., 2016b). However, monounsaturated fatty acids (MUFAs) are synthesized by SCD, and induce a ferroptosis-resistant state in ovarian cancer (Tesfay et al., 2019) and lung cancer cells (Wohlhieter et al., 2020) by reducing the accumulation of cytotoxic lipid ROS in the biomembrane.

**FIG. 7.**
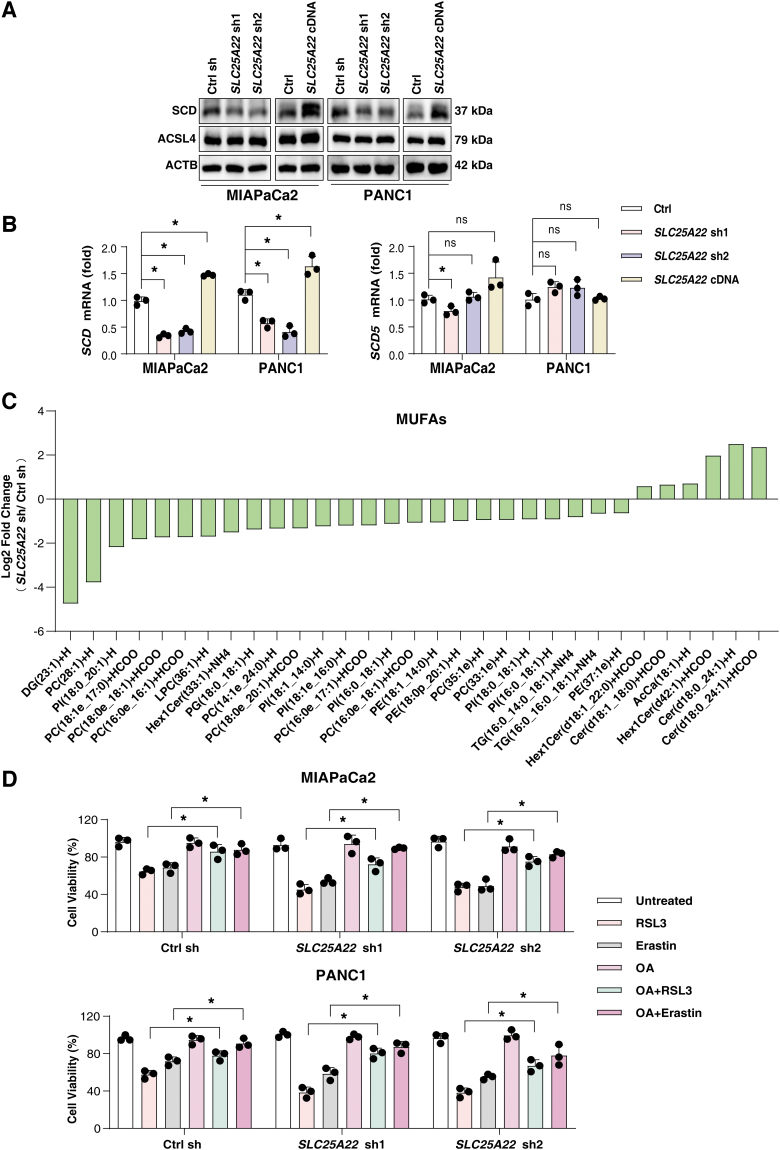
**SLC25A22 upregulates the expression of SCD. (A)** Analysis of SCD and ACSL4 protein expression in indicated PDAC cells. **(B)** Analysis of SCD and SCD5 expression in indicated PDAC cells. **(C)** Changes in the phospholipid compositions in PANC1 cells after SLC25A22 knockdown. **(D)** Cell viability of indicated PDAC cells after treatment with RSL3 (1 μ*M*) or erastin (20 μ*M*) in the absence or presence of OA (50 μ*M*) for 24 h. Data in **(B, D)** are presented as the mean ± SD of three technical replicates from one representative dataset of three independent experiments. Statistical significance was analyzed using two-way ANOVA with Dunnett's *post hoc* test. *p < 0.05. ns, not significant. ACSL4, acyl-CoA synthase long-chain family member 4; OA, oleic acid; SCD, stearoyl-CoA desaturase.

To explore the effect of SLC25A22 on lipid peroxidation, we detected the expression of ACSL4 and SCD in SLC25A22-knockdown or overexpressed PDAC cell lines. Our data demonstrated that SCD positively correlated with the expression of SLC25A22, whereas the expression of ACSL4 was not significantly altered in PANC1 and MIAPaCa2 cells (Fig. 7A). Earlier studies have shown that stearoyl-CoA desaturase 5 (SCD5) has a desaturation capacity similar to that of SCD in humans (Bellenghi et al., 2015). However, SLC25A22 did not affect the gene expression of SCD5, but instead had an effect on SCD expression in PANC1 and MIAPaCa2 cells (Fig. 7B).

Considering that SCD is an enzyme that converts saturated fatty acids to MUFAs (Paton and Ntambi, 2009), we hypothesized that SLC25A22-mediated ferroptosis resistance might be associated with SLC25A22-mediated SCD expression. To further investigate the underlying mechanism of SLC25A22 in ferroptosis, we performed a liquid chromatography–mass spectrometry (LC-MS)–based nontargeted lipidomic analysis to determine changes in lipid composition upon depletion of SLC25A22 in PANC1 cells. This assay showed that SLC25A22 knockdown significantly reduced levels of 25 MUFAs (FC <0.67, *p* < 0.05; Fig. 7C).

**FIG. 8.**
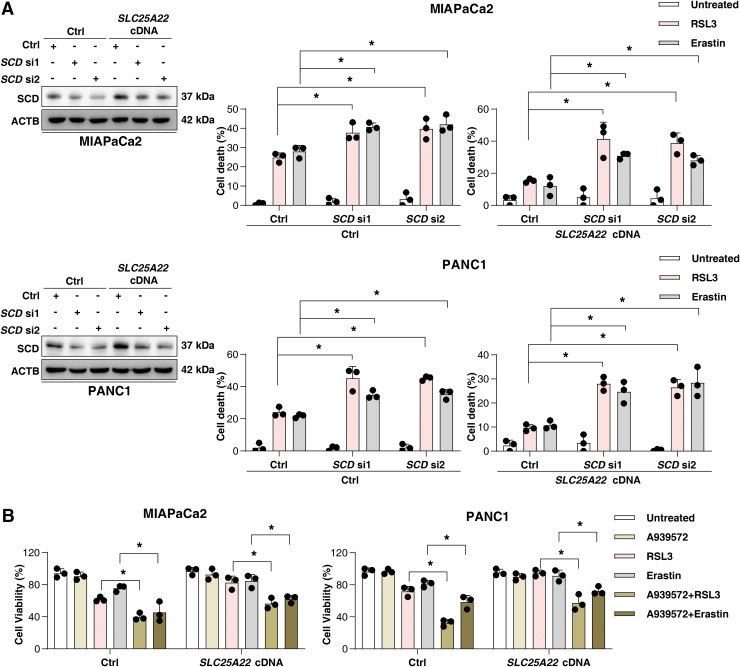
**SLC25A22 upregulates the expression of SCD. (A)** Analysis of SCD protein expression in indicated PDAC cells (*left*). Cell viability of indicated PDAC cells after treatment with RSL3 (1 μ*M*) or erastin (20 μ*M*) for 24 h (*right*). **(B)** Cell viability of indicated PDAC cells after treatment with RSL3 (1 μ*M*) or erastin (20 μ*M*) in the absence or presence of A939572 (5 μ*M*) for 24 h. Data in **(A, B)** are presented as the mean ± SD of three technical replicates from one representative dataset of three independent experiments. Statistical significance was analyzed using two-way ANOVA with Dunnett's *post hoc* test. *p < 0.05.

MUFAs can inhibit ferroptosis (Magtanong et al., 2019), providing a mechanistic explanation for our observation. Indeed, the supplementation with MUFA oleic acid (OA; 18:1) improved cell growth after RSL3 or erastin treatment in PDAC (Fig. 7D). The downregulation of SCD using siRNA or the SCD inhibitor A939572 sensitized SLC25A22-overexpressed PDAC cells to RSL3-induced ferroptosis (Fig. 8A, B). Collectively, we demonstrated that SLC25A22 promotes MUFA production and ferroptosis resistance by expressing SCD in PDAC cell lines.

### SLC25A22 upregulates the SCD expression in an AMPK-dependent manner

As a mitochondrial glutamate transporter, SLC25A22 maintains the integrity of mitochondrial function (Ruprecht and Kunji, 2020). Previous studies have shown that the knockdown of SLC25A22 reduces ATP production through the TCA cycle in colorectal cancer cells (Wong et al., 2016). Our study revealed that the overexpression of SLC25A22 inhibited ATP depletion induced by the ferroptosis inducers RSL3 or erastin (Supplementary Fig. S1D). Furthermore, the knockdown of SLC25A22 enhanced ATP depletion induced by RSL3 or erastin (Supplementary Fig. S1D). Therefore, SLC25A22 is an important regulator of intracellular ATP levels during ferroptosis.

**FIG. 9.**
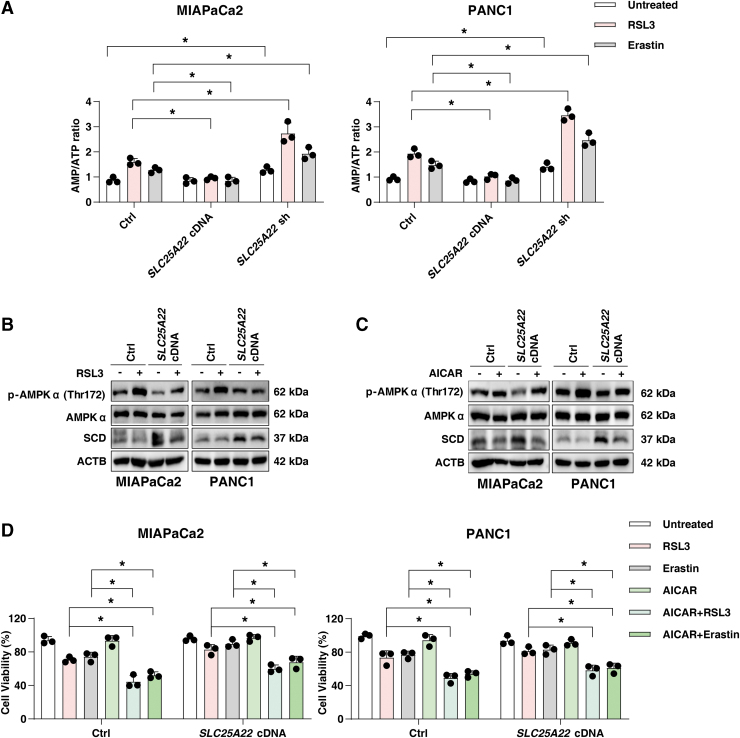
**AMPK is required for SLC25A22-mediated SCD expression. (A)** The indicated PDAC cells were treated with RSL3 (1 μ*M*) or erastin (20 μ*M*) for 24 h, and AMP/ATP levels were measured. **(B)** Analysis of protein expression in indicated PDAC cells after treatment with RSL3 (1 μ*M*) for 24 h. **(C)** Analysis of protein expression in indicated PDAC cells after treatment with AICAR (200 μ*M*) for 24 h. **(D)** Cell viability of indicated PDAC cells after treatment with RSL3 (1 μ*M*) or erastin (20 μ*M*) in the absence or presence of AICAR (200 μ*M*) for 24 h. Data in **(A, C)** are presented as the mean ± SD of three technical replicates from one representative dataset of three independent experiments. Statistical significance was analyzed using two-way ANOVA with Dunnett's *post hoc* test. *p < 0.05. AICAR, 5-aminoimidazole-4-carboxamide ribonucleotide; AMPK, AMP-activated protein kinase; ATP, adenosine triphosphate.

The energy sensor AMPK can be activated in cells with increased AMP/ATP ratio (Lin and Hardie, 2018), and plays a dual role in ferroptosis depending on stress conditions and cell type (Kang et al., 2018; Lee et al., 2020; Song et al., 2018). Specifically, AMPK-mediated downregulation of SCD promotes ferroptosis in hepatocellular carcinoma cells by inhibiting MUFA production (Zhao et al., 2020). The overexpression of SLC25A22 suppressed the increase in AMP/ATP ratio induced by RSL3 or erastin (Fig. 9A and Supplementary Fig. S1E). The knockdown of SLC25A22 further enhances the AMP/ATP ratio induced by RSL3 or erastin in PDAC cells (Fig. 9A and Supplementary Fig. S1E).

Western blot analysis revealed that RSL3 induced p-AMPK (Thr172) expression in wild-type PDAC cells, whereas overexpression of SLC25A22 inhibited this process (Fig. 9B). Moreover, the AMPK agonist 5-aminoimidazole-4-carboxamide ribonucleotide (AICAR) inhibited the expression of SCD in SLC25A22-overexpressed PDAC cells (Fig. 9C), and enhanced the anticancer activity of RSL3 or erastin in SLC25A22-overexpressed MIAPaCa2 and PANC1 cells (Fig. 9D). Taken together, these results suggest that SLC25A22 upregulates the expression of SCD in an AMPK-dependent manner, thereby inhibiting ferroptosis by promoting the production of MUFAs.

### SLC25A22 inhibits the anticancer activity of RSL3 *in vivo*

**FIG. 10.**
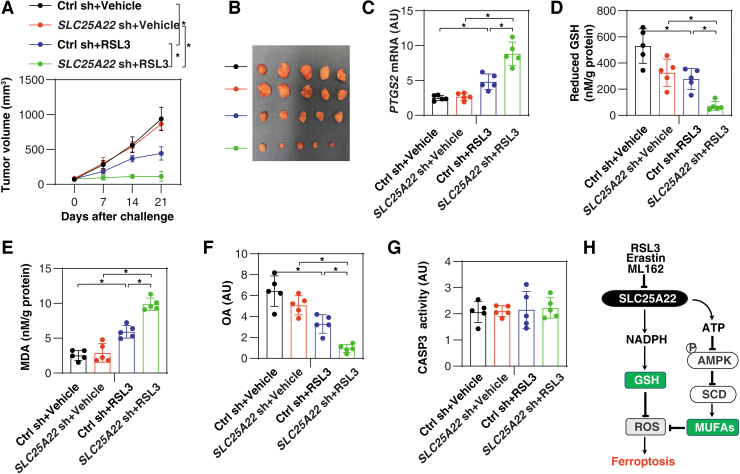
**SLC25A22 inhibits the anticancer activity of RSL3 *in vivo*. (A)** NOD SCID mice were injected subcutaneously with the indicated PANC1 cells (5 × 10^6^/mouse) and treated with RSL3 (100 mg/kg, once every other day, intratumoral injection) at day 7 for 2 weeks. Tumor volume was calculated weekly (*n* = 5 mice/group; *p < 0.05, ANOVA with Tukey's multiple comparisons test; data are presented as means ± SD). **(B)** Photographs of isolated tumors at day 21. **(C–G)** In parallel, the activity or levels of PTGS2 mRNA **(C)**, reduced GSH **(D)**, MDA **(E)**, OA **(F)**, and CASP3 activity **(G)** in isolated tumors at day 21 were assayed (*n* = 5 mice/group; *p < 0.05, ANOVA with Tukey's multiple comparisons test; data are presented as means ± SD). **(H)** A model illustrating that SLC25A22 blocks ferroptosis by producing GSH and MUFA. CASP3, caspase 3; MUFA, monounsaturated fatty acid; PTGS2, prostaglandin-endoperoxide synthase 2.

To evaluate the effects of SLC25A22-mediated ferroptosis resistance *in vivo*, we used a PANC1-derived xenograft model in immunodeficient NOD SCID mice (Liu et al., 2021b). Compared with the control group, the anticancer activity of RSL3 was increased in the SLC25A22-knockdown (*SLC25A22* KD) PANC1 group (Fig. 10A, B). Subsequent analysis of ferroptosis marker prostaglandin-endoperoxide synthase 2 (Fig. 10C) (Chen et al., 2021a), GSH (Fig. 10D), MDA (Fig. 10E), and MUFA OA levels (Fig. 10F) confirmed the role of SLC25A22 in inhibiting RSL3-induced ferroptosis in isolated tumor. In contrast, the activity of caspase 3 (CASP3) was not changed in the *SLC25A22* KD group in the absence or presence of RSL3 (Fig. 10G). These animal studies further demonstrated the selective negative regulation of SLC25A22 in ferroptotic cancer death *in vivo*.

## Discussion

The induction of ferroptosis is closely linked to metabolic dysregulation, resulting in unlimited lipid peroxidation (Stockwell et al., 2017). In this study, we analyzed changes in the SCL25 family in ferroptosis and identified mitochondrial transporter SLC25A22 as a previously unidentified suppressor of ferroptosis in human PDAC cells. Specifically, we demonstrated that two metabolic pathways (the production of GSH and the synthesis of MUFAs) contribute to SLC25A22-mediated ferroptosis resistance (Fig. 10H). Our findings also reinforced the notion that mitochondria are the hub integrating multiple metabolic pathways to control cell survival and death (Martinez-Reyes and Chandel, 2020).

The process of ferroptosis is associated with many genetic changes and protein abnormalities (Xie et al., 2016). We focused on SLC25 family members because of their context-sensitive roles in transporting metabolites and other molecules to mitochondria. An impaired function of the SLC25 family leads to compartmentalized accumulation of substrates, leading to impaired mitochondrial metabolism and the development of various diseases (Kunji et al., 2020).

Although the exact transcriptional regulatory mechanism remains undetermined, our study shows that the most affected SLC25 family gene, SLC25A22, is selectively downregulated in four PDAC cell lines in response to multiple ferroptotic activators rather than classical apoptotic stimuli. Whether SLC25A22 expression is a biomarker of ferroptosis sensitivity requires further investigation in other cell lines or tissues.

SLC25A22 exerts oncogenic effects in colorectal cancer (Wong et al., 2016), gallbladder cancer (Du et al., 2019), and osteosarcoma cells (Chen and Wu, 2018), mainly through its transport of glutamate across the inner mitochondrial membrane to the mitochondrial matrix. Glutamine is the most abundant free amino acid in the circulation that helps rapidly proliferating cells (including cancer cells) meet increasing demands for ATP, biosynthetic precursors, and reducing agents (Asantewaa and Harris, 2021).

Glutamate can be converted to the TCA cycle intermediate α-KG by various mechanisms in the mitochondria, which further initiates citrate-dependent lipid synthesis in the cytosol (Maus and Peters, 2017). We demonstrated that SLC25A22 contributes to the production of NADPH, which is used as a cofactor by GSH reductase to reduce oxidized GSH. GSH, together with NADPH-producing pathways and GSH reductase, provides a defense system against oxidative damage during ferroptosis (Kuang et al., 2020).

Lipid peroxidation is the core hallmark of ferroptosis (Tang and Kroemer, 2020). PUFA is one of the main targets of lipid peroxidation, and it appears that extensive oxidation of PUFA-containing phospholipids might modify membrane structure and increase membrane permeability, eventually resulting in plasma membrane rupture in response to the accumulation of oxidized lipids (Li et al., 2019). In contrast to PUFAs, exogenous MUFAs decrease oxidized lipid accumulation and inhibit ferroptosis in an acyl-CoA synthetase long-chain family member 3-dependent manner (Magtanong et al., 2019). SCD, an enzyme that catalyzes the rate-limiting step in MUFA synthesis, is upregulated under stress in cancer cells (Paton and Ntambi, 2009; Wohlhieter et al., 2020).

A previous study showed that SCD protects cells from ferroptosis through desaturating fatty acids and tumor endothelial cell-derived fatty acid binding protein 4 to enhance lipid droplet formation in cancer cells (Luis et al., 2021). SCD-mediated lipogenesis is downstream of the PI3K–AKT–MTOR pathway, which suppresses ferroptosis in cancer cells (Yi et al., 2020). Taken together, our findings, combined with these independent studies by others, underscore the important role of SCD in blocking ferroptosis.

AMPK is a major energy sensor in lipid and glucose metabolism, and controls many physiological and pathological processes, including cell death (Xiao et al., 2013). AMPK-mediated BECN1 phosphorylation at Ser90/93/96 promotes ferroptosis in human colon cancer cells by inhibiting the production of intracellular GSH (Song et al., 2018). AMPK activation can also inhibit ferroptosis by blocking fatty acid synthesis *via* the phosphorylation of acetyl-CoA carboxylase (Lee et al., 2020). Recent studies show that SCD controls lipid biosynthesis and cell growth through AMPK pathway regulation (Dziewulska et al., 2020; Zhu et al., 2019). Therefore, AMPK may play a dual role in ferroptosis, depending on its substrates and signaling pathways.

Our results indicate that an excessive activation of SLC25A22 inhibits the phosphorylation of AMPK, which leads to the upregulation of SCD. We also demonstrated that treatment with the AMPK agonist AICAR reduces cell viability in SLC25A22-overexpressed PDAC cells. These results suggest that SLC25A22 upregulated SCD expression in an AMPK-dependent manner, thereby promoting ferroptosis resistance.

## Conclusion

We established a novel ferroptosis defense pathway that relies on the mitochondrial glutamate transporter SLC25A22 to mediate the product of GSH and MUFAs and subsequently detoxify lipid peroxides. Further understanding the expression, function, and regulation of mitochondrial glutamate transporters may provide new therapeutic targets for the treatment of PDAC and other types of cancer.

## Materials and Methods

### Reagents

The antibodies to ACTB (66009-1-Ig), PARP1 (13371-1-AP), DHODH (14877-1-AP), G6PD (25413-1-AP), and solute carrier family 11 member 2 (SLC11A2, also known as DMT1; 20507-AP) were obtained from Proteintech Biotechnology. The antibodies to SLC25A22 (ab137614), GPX4 (ab125066), solute carrier family 7 member 11 (SLC7A11; ab216876), ACSL4 (ab155282), and NCOA4 (ab86707) were purchased from Abcam. The antibodies to SCD (A16429) were obtained from ABclonal.

The antibodies to AMPKα (5831) and phospho-AMPKα (Thr172; 2535) were obtained from Cell Signaling Technology. RSL3 (S8155), ferrostatin-1 (S7243), liproxstatin-1 (S7699), necrostatin-1 (S8037), Z-VAD-FMK (S7023), erastin (S7242), STS (S1421), OA (S4707), and AICAR (S1802) were purchased from Selleck Chemicals. A939572 (T4515) was purchased from TOPSCIENCE. Oxalacetic acid (328-42-7) was purchased from MACKLIN. GSH monoethyl ester (353905) and NAC amide (A0737) were purchased from MilliporeSigma. BODIPY 581/591 C11 (D3861) was obtained from Thermo Fisher Scientific.

### Cell culture and treatment

SW1990 (CRL-2172), MIAPaCa2 (CRL-1420), PANC1 (CRL-1469), and ASPC1 (CRL-1682) cell lines were obtained from the American Type Culture Collection. These cells were grown in Dulbecco's modified Eagle's medium or Eagle's minimum essential medium with 10% fetal bovine serum (12A288), 2 m*M* of l-glutamine, and 100 U/mL of penicillin and streptomycin at 37°C in a humidified atmosphere of 5% CO_2_.

Cell line identity was validated by short tandem repeat profiling, and routine mycoplasma testing was negative for contamination. Dimethyl sulfoxide (DMSO, IC0219605525; VWR International) was used to prepare the stock solution of ferroptosis inducers. The final concentration of DMSO in the drug working solution in the cells was <0.01%. DMSO of 0.01% was used as a vehicle control in all cell culture assays (Chen et al., 2022a).

### Western blot assay

Cells were lysed in RIPA Lysis Buffer (BL504A; Biosharp) containing phenylmethylsulfonyl fluoride (BL507A; Biosharp) on ice for 30 min and vortexed every 10 min. After centrifugation at 16,000 *g* for 15 min at 4°C, the supernatants were collected and quantified using BCA assay (23225; Thermo Fisher Scientific) (Dai et al., 2020b; Kang et al., 2011; Liu et al., 2021c). Then, 30 μg of each sample was resolved on 10% PAGE gels (PG113; EpiZyme Biotechnology) and transferred to a polyvinylidene difluoride membrane.

After blocking with 5% milk at room temperature for 2 h, the membrane was incubated overnight at 4°C with various primary antibodies (1:500–1:1000). After incubation with peroxidase-conjugated secondary antibodies for 1 h at room temperature, the signals were visualized using SuperSignal West Pico PLUS Chemiluminescent Substrate (34580; Thermo Fisher Scientific) and by using the ChemiDoc Touch Imaging System (Bio-Rad).

### qPCR assay

Total RNA was extracted and purified from cultured cells using a Cell Total RNA Isolation Kit (RE-03112; ForeGene). First-strand cDNA was synthesized from 1 μg of RNA using the PrimeScript RT Master Mix (RR036A; Takara). Then, cDNA from various cell samples was amplified using qPCR with specific primers as described in Table 1. Optimal dilution and melting curves were utilized to ensure the specificity of amplified production for each primer set. The date was normalized to GAPDH mRNA, and the fold change (FC) was calculated using the 2^−ΔΔCt^ method (Wang et al., 2020). Relative concentrations of mRNA were expressed in arbitrary units based on the untreated group, which was assigned a value of 1 (Deng et al., 2018).

**Table 1.** Reverse Transcription-Quantitative Polymerase Chain Reaction Primer Design and Synthesis

Gene	Forward sequence	Reverse sequence
SLC25A1	GTGAAGTTCATCCACGACCAGAC	TGCTTCAGGACAGTGGCTGTGA
SLC25A2	CTGCTTCCTGAAGACATACGCC	CACTTTCCTGACAAACTGCTGGC
SLC25A3	CTGGCTCCTATGGAAGCTGCTA	GTCTCATCCAGAGAGGAGCAAC
SLC25A4	GCTGCCTACTTCGGAGTCTATG	TGCGACTGCCGTCACACTCTG
SLC25A5	TCCAAGATGGCTGTAGTGCCCA	AAGTTACCGCGCCAGAAGGACA
SLC25A6	GGTGAAGATCACCAAGTCCGAC	ACCACGATGTGCGTGTTCTTGG
UCP1	AGTTCCTCACCGCAGGGAAAGA	GTAGCGAGGTTTGATTCCGTGG
UCP2	TGGTCGGAGATACCAAAGCACC	GCTCAGCACAGTTGACAATGGC
UCP3	GACTATGGACGCCTACAGAACC	CTCCTTGAGGATGTCGTAGGTC
SLC25A10	GCAGACTTGGTCAACGTCAGGA	CATGGTTGCACCCGAGAACAGT
SLC25A11	ATCAGCGGTCTTGTCACCACTG	GCGGACAACTTTGAACAGCACG
SLC25A12	GGGCTTCTTTGGACTCTACAGG	GGAAGTGGAACAGAGCCATCTC
SLC25A13	AGATGGTTCGGTCCCACTTGCA	ACCAGTGGTGATTTCTCCTGCC
SLC25A14	TCGAATGCAGGCTCAAGGAAGC	ACTCCTACAACGATGGCAGCAC
SLC25A15	GGAGACATCAGGGAAGATAGCC	GCTCAGTTCATAGCCACCGAAG
SLC25A16	ATGCTCCTACCCTTCTTGGCAG	TTGCATTCGCCGACGAGTCACA
SLC25A17	GGTGGTAAACACCAGACTGAAGC	AGCCGAGATTCCTTCATCGCGA
SLC25A18	GCAGTGAACCTCACTCTGGTCA	GGCAAGCATCTCCATCTTCAGG
SLC25A19	GCCATACCAGCCGAAGGAAAGA	CTCCAACCTGTAGCCGCTTCTT
SLC25A20	ACCGAGTTTGCCTGGACAACCT	CCCAAAGAAGCACACGGCAAAC
SLC25A21	GATTCAGAGATGTGCAACCGATC	GGTTTCAGCCAAGATAGGTGGC
SLC25A22	GTCAACGAGGACACCTACTCTG	GGAAGTAGACCACCTGTGCGAT
SLC25A23	CTCTGGGCATTTCCATCTCGCT	CAGCGAATGCAACAGGAAGTGG
SLC25A24	GGCTTTGGTGAGAACTCGCATG	GGTGATGCCTCTGTAAAGTCCTG
SLC25A25	GCATCGTTGGTGGCTTCACTCA	CCTCAGAGTCTCCTGGTCACTA
SLC25A26	CAGCAGTCTGTGGAGCTTTTGC	TGCAGGACAGAGAGCACATTCC
SLC25A27	TGGGAACACCAGCCGATGTCAT	GAACAGCCTGAATCAAGCAGTCA
SLC25A28	AGCCACTGTCACCACGCACATG	GTCAGGCTGTAGACTCTGCATC
SLC25A29	AGAGCGTGGAGAAGCCTCAGTA	CGTTGATGAAGGTGAGCCCCAT
SLC25A30	GATACCGAGGAATGTTGCACGC	CCATAGGATGCCTGGCGTAACA
SLC25A31	CGGTACAAAGGCATGGTGGACT	CTCCAGACATGAATAGCTGCTTG
SLC25A32	GCGTCTTATCCAACCTTGCGCT	GTTTCCAAATGGTAGTCAAGCAATG
SLC25A33	CGGACAGTCTACTATCCTCAGG	GTGACTTTGGTCCCTCTTTCTCC
SLC25A34	GCCACCTTCGCCTCTGCCAAG	TGACGACAACCACGGCTATGCT
SLC25A35	TGCACAAGGCTCTACAACCAGC	CCAGTAAAGGCGCTGAAGCACT
SLC25A36	GCTGCTTATTCAAACTGCAAGGAA	CCTTGCATCAAGCTGTAACCGAG
SLC25A37	CCACATGACAGCAGGAGCGATG	CTTGTGTACTGGGCTTTGGGATC
SLC25A38	GTTGGCTGTACTCTTGAAGGTGG	GTGCCAAAGTAGATTCCAACGCC
SLC25A39	CACTGCCTATGACCAACTGAAGG	CTTTGTCCGCATAAGCTCCAGG
SLC25A40	GCCTCATGTACTGGAGCTATACT	TGTTGCCTCCCTCTTCACAGAC
SLC25A41	CTGGAAGTGGATAACAAGGAGGC	GGTGAAGTTCGTCTTGGAGGAG
SLC25A42	AGTTCAGCGCACACGAGGAGTA	GTAGGTCAGTGAAGCGGCTGTC
SLC25A43	TTCTCAGGAGCAGTGGACTGCT	TCTCTTGCAGAACTCAAAGGTGC
SLC25A44	GAGGCTATGTGGCTTCACTGCT	GACAATGTGAGGGCACTCCTTAG
SLC25A45	ACCCGTTTGACACTGTAAAGGTG	CAGGACAGAGTTGACCACAGCT
SLC25A46	GGAGTCACACTTGGAGCAGAAG	GGATTTCAGTAGAAGGTGTTCTCC
SLC25A47	ACGGAGCCAAAGTACACAGGCA	GTAGGTGCCAAAAGACACGGAAG
SLC25A48	CACCCTCTGGACACAGTCAAGA	TTGTAGACGGCAATGCTGGCGA
MTCH1	ACTGAGGCTCTTTTCGTGGCAC	TCGGCAGATAGAGGACCTTCCT
MTCH2	GCAAGTGTGTCAGCTTCCTGGT	TGGACCACAGTTCCAAGGACTC
SLC25A51	TACCGCAACGACTCACAGTGCT	CCACCAATCTGAGACTGTATGCG
SLC25A52	ATCTGCCTACCGCAACGACTCA	CCACCAATCTGAGACTGTAGGC
SLC25A53	AGGCTGTGAGACAGCTTTGGCA	GAGAGAAAGCACAGCAGGCTATC
SCD	CCTGGTTTCACTTGGAGCTGTG	TGTGGTGAAGTTGATGTGCCAGC
SCD5	GAGGAATGTCGTCCTGATGAGC	GCCAGGAGGAAGCAGAAGTAGG
PTGS2	CGGTGAAACTCTGGCTAGACAG	GCAAACCGTAGATGCTCAGGGA

### RNA interference

Human SLC25A22 cDNA (sequence: CCAACTTTGTGCCAACCGGTCGCCACCATGGCTGATAAGCAGATCAG and AATGCCAACTCTGAGCTTGGCCTGGGGGTCCTGCAGCAGCC) and control cDNA were obtained from Gene Biotechnology. Human SLC25A22 shRNA-1 (sequence: GCCAACGACTTCTTCCGACAT), human SLC25A22 shRNA-2 (sequence: GTCGCCTTTCTACGTGTCCTT), and control shRNA (pLKO.1) were obtained from Sigma-Aldrich. Human DHODH siRNA-1 (sequence: GCCACGGGAGAUGAGCGUUTT) and human DHODH siRNA-2 (sequence: CCUUCCACGGGCCAGAUUUTT) were obtained from GenePharma.

Human SCD siRNA-1 (sequence: GGUUGAAUAUGUCUGGAGA) and human SCD siRNA-2 (sequence: GCUAGACUUGUCUGACCUA) were obtained from IGEbio. RNAi testing was performed using Lipofectamine 3000 (Thermo Fisher Scientific) according to the manufacturer's instructions. Stable knockdown cells were selected by adding puromycin (Liu et al., 2021c).

### Cell viability and cell death assays

The level of cell viability was assayed using a CCK8-Kit (40203ES80; Yeasen Biotechnology) according to the manufacturer's protocol. The absorbance values were measured at 450 nm. A Hoechst 33342/Propidium Iodide Cell Death Assay Kit (BB-4131-1; BestBio Biotechnology) was used to assay cell death according to the manufacturer's protocol. The percentage of cell death was determined by confocal microscopy from 10 random fields (Lin et al., 2022).

### Lipid peroxidation assay

The relative MDA concentration in cell lysates was assessed using a Lipid Peroxidation MDA Assay Kit (S0131; Beyotime) according to the manufacturer's instructions (Dai et al., 2020a; Kuang et al., 2021). C11-BODIPY (5 μ*M*) was added to drug-treated and untreated cells for 0.5 h, then the cells were washed with phosphate-buffered saline (PBS) and collected by trypsin. Oxidation of the polyunsaturated butadienyl portion of C11-BODIPY resulted in a shift of the fluorescence emission peak from ∼590 to ∼510 nm. Cells were analyzed using flow cytometry after washing twice with PBS, and the results were analyzed with Flowjo 10 software.

### Apoptosis assay

After treatment with the indicated compounds, the cells were stained using an Annexin V-FITC Apoptosis Detection Kit (C1062L; Beyotime) according to the manufacturer's protocol. Samples were analyzed on a BD flow cytometer, and data were analyzed with FlowJo 10.0 software. The activity of CASP3 in tumor samples was assayed using an enzyme-linked immunosorbent assay (ELISA) Kit (5723) from Cell Signaling Technology according to the manufacturer's protocol.

### Cytosolic and mitochondrial glutamate measurement

We isolated mitochondria and cytosolic fractions using the Cell Mitochondrial Isolation Kit (C3601; Beyotime) according to the manufacturer's instructions. In brief, designated PDAC cells were harvested and incubated with cold mitochondrial isolation reagent for 15 min. Cells were homogenized and centrifuged at 1000 *g* for 10 min to obtain the supernatant. The supernatant was then centrifuged at 11,000 *g* for 10 min. Cytosolic and mitochondrial glutamate levels were measured using the Glutamate Assay Kit (EH6963M; WELLBIO) according to the manufacturer's instructions.

### Sample preparation and data acquisition for metabolomics

The culture medium was aspirated, and the cells were washed twice in ice-cold PBS and once in ice-cold saline (0.9% NaCl). We then added a small amount of ice-cold PBS to the dish, scraped off the cells, and transferred them to a new 1.5 mL Eppendorf tube. The mixture was centrifuged for 1 min at 1000 *g* at 4°C. Cells were stored at −80°C after cooling with liquid nitrogen and used for subsequent analysis.

The cell samples were vortexed for 30 s with 1 mL of acetonitrile:methanol:ddH_2_O mixed solution (2:2:1, v/v/v). Then, the samples were placed in a high-throughput tissue grinder for 2 min at 60 Hz, and placed into liquid nitrogen for 5 min and thawed at room temperature, with the operation being repeated twice. The mixture was centrifuged at 4°C for 10 min at 13,000 rpm, and 850–900 μL of the supernatant was transferred from each sample into another 2 mL centrifuge tube. Samples were dried in a vacuum and dissolved with 300 μL of 2-chlorobenzalanine solution (4 ppm) prepared with acetonitrile: 0.1% FA (1:9, v/v; −20°C), then the supernatant was filtered through a 0.22 μm membrane to prepare the samples for LC-MS. Next, 35 μL of each sample was reserved for quality control, and the remainder was used for LC-MS detection.

Chromatographic segregation was achieved with an ACQUITY UPLC HSS T3 (150 × 2.1 mm, 1.8 μm; Waters, Inc.) column maintained at 40°C. The temperature of the autosampler was 8°C. The mobile phases were water (A) and acetonitrile (B), both with 0.1% formic acid. The injection volume was 2 μL. Data were acquired in both positive and negative ion mode.

### Data processing and pathway analysis

A *p* value variable importance projection (VIP; produced by OPLS-DA) and FC were used to discover the contributable variables for classifications. A *p* value of <0.05 and VIP value of >1 were used to identify statistically significant metabolites. Differential metabolites were subjected to pathway analysis by MetaboAnalyst, which combines results from powerful pathway enrichment analysis with a pathway topology analysis. The identified metabolites in metabolomics were then mapped to the Kyoto Encyclopedia of Genes and Genomes (KEGG) pathway for biological interpretation of higher level systemic functions. The metabolites and corresponding pathways were visualized using a KEGG Mapper tool.

### NADP^+^/NADPH measurements

The intracellular rates of NADP^+^ and NADPH were measured using an NAD^+^/NADH Assay Kit with WST-8 (S0175; Beyotime) according to the manufacturer's instructions. In brief, 1 × 10^6^ indicated that PDAC cells were collected and lysed with 200 μL of NADP^+^/NADPH extraction solution. Lysate samples were centrifuged at 12,000 *g* for 8 min at 4°C to collect the supernatant. The supernatant was then separated into two portions. One portion was heated to 60°C to deplete NADP^+^. Together with G6PDH working solution, the samples were added to 96-well plates and incubated at 37°C for 10 min under dark conditions. The absorbance was then measured at 450 nm.

### ATP and AMP measurements

The content of ATP was measured using an ATP Bioluminescence Detection Kit (S0026; Beyotime) according to the manufacturer's instructions. In brief, the indicated PDAC cells were extracted and pyrolyzed with a lysis buffer supplied with the kit. The lysate was centrifuged at 12,000 *g* for 5 min at 4°C. Furthermore, 20 μL of supernatant and 100 μL of ATP detection buffer were mixed, and the luminescence was measured using a microplate reader. ATP standard solutions (0, 0.5, 1, 5, 10, and 50 μ*M*) were used to obtain a standard curve.

The level of AMP was measured using a human-specific ELISA kit (21601; Jiangsu Meibiao Biotechnology Co., Ltd.) according to the manufacturer's instructions. Protein concentration of the supernatant was measured using the BCA Protein Assay Kit (NCI3225CH; Thermo Fisher Scientific). ATP levels were calculated according to the standard curve and normalized against the standard's protein concentration.

### GSH and intracellular iron analyses

Intracellular total GSH levels in PDAC cell lysates were measured using a GSH/GSSG Assay Kit (S0053; Beyotime) according to the manufacturer's instructions. In brief, 10 μg of collected cell samples were dissolved in 30 μL of M solution. The levels of GSH and GSSG were measured using a microplate reader with absorption at 412 nm. GSSG levels were calculated by subtracting GSH from the total GSH levels. Intracellular iron content was detected using the green fluorescent Fe^2+^ indicator Phen Green SK, Diacetate (P14313; Thermo Fisher Scientific) according to the manufacturer's instructions. In brief, the indicated PDAC cells were seeded in six-well plates. After staining with 10 μ*M* Phen Green SK, the plates were incubated at 37°C and 5% CO_2_ for 30 min. Morphological changes were examined by fluorescence microscopy (Yu et al., 2015).

### Lipidomic analyses

Indicated PANC1 cells (1 × 10^7^) were collected in a 2-mL centrifuging tube. Lipids were extracted according to a methyl tertiary butyl ether (MTBE) method. In brief, samples were homogenized with 0.2 mL 4°C water and 0.24 mL precooled methanol. Then, the samples were vortexed and mixed with 800 μL of MTBE. The mixture was exposed to ultrasound for 20 min at 4°C. The samples were kept at room temperature for 30 min before being centrifuged at 14,000 *g* for 15 min at 10°C. The upper organic solvent layer was collected and dried under nitrogen. The samples were stored at −80°C until employed for LC-MS lipidomic analysis.

Chromatographic segregation was accomplished using a UHPLC Nexera LC-30A system carrying the CSH C18 column (1.7 μm, 2.1 × 100 mm; Waters, Inc.), in which the temperature was set at 45°C. The lipid extracts were redissolved in 200 μL of 90% isopropanol/acetonitrile, centrifuged at 14,000 *g* for 15 min, and finally 3 μL of sample was injected. Solvent A consisted of acetonitrile/water (6/4, v/v) with 0.1% formic acid and 0.1 m*M* ammonium formate; solvent B consisted of acetonitrile/isopropanol (1/9, v/v) with 0.1% formic acid and 0.1 m*M* ammonium formate.

The initial mobile phase included 30% of solvent B at a flow rate of 0.3 mL/min. It was held for 2 min, and then linearly increased to 100% solvent B in 23 min, followed by equilibrating at 5% solvent B for 10 min. Mass spectra were acquired by Q-Exactive Plus in positive and negative mode, respectively. Electrospray ionization parameters were optimized and preset for all measurements as follows: source temperature, 300°C; capillary temp, 350°C; ion spray voltage, 3000 V; S-lens RF level, 50%; and a scan range of the instruments, *m/z* 200–1800.

A lipid search engine was used for the identification of lipid species based on MS/MS math. A univariate analysis (*t-*test) was used to calculate the statistical significance (*p* value). Metabolites with a VIP score of >1, *p* value of <0.05, and FC analysis of >1.5 or <0.67 were considered differentially expressed (Lin et al., 2022; Song et al., 2021).

The lipidomic data have been deposited to the ProteomeXchange Consortium (http://proteomecentral.proteomexchange.org) *via* the iProX partner repository (Chen et al., 2022b) with the dataset identifier PXD040701.

### OA preparation

We mixed 28.264 mg of OA (S4707; Selleck Chemicals) with 25 mL of 0.1 *M* sodium hydroxide solution. The mixture was then placed in a 70°C water bath for 45 min until the mixture became clear and filtered. The solution was then immediately placed into 25 mL of 10% fatty acid-free bovine serum albumin (36104ES25; Yeasen).

### Animal models

Control or SLC25A22 KD PANC1 cells (5 × 10^6^ cells) were injected subcutaneously into the dorsal side of NOD SCID mice (female, 8–10 weeks old). On the seventh day, these mice were given RSL3 (100 mg/kg, once every other day, intratumoral injection) for 2 weeks as previously described (Yang et al., 2014). The diameter of the tumor was measured twice a week with a caliper, and the tumor volume was calculated using the following formula: length × width^2^ × π/6. At 21 days, the mice were euthanized, and the xenograft solid tumors were collected.

All mice were maintained under specific pathogen-free conditions on a regular 12-h light and dark cycle (7:00–19:00 light period; room temperature 20°C–25°C; relative humidity: 40%–60%). Food and water were available *ad libitum*. All animal experiments were conducted in accordance with the institutional ethics guidelines related to animal care, and were approved by an institutional animal health and use committee.

### Statistical analysis

The data were presented as the mean ± standard deviation of three technical replicates from one representative dataset of three independent experiments, unless otherwise indicated. We performed statistical analyses using GraphPad Prism 8 software with the support of our institutional statistical core. Generally, we used standard unpaired Student's *t*-test to compare two independent groups. Analysis of variance (ANOVA) was used to test for differences among three or more groups, and if the ANOVA showed significant differences we used Dunnett's or Tukey's *post hoc* test to identify pairs with significant differences.

The statistical methods used in each experiment are listed in the corresponding figure legends. A *p* value of <0.05 was considered statistically significant. We did not exclude samples or animals. Our animal experiments were not blinded. No statistical methods were used to predetermine sample sizes, but our sample sizes are similar to those generally employed in the field. Electronic laboratory notebook was not used.

## Supplementary Material

Supplemental data

## Abbreviations Used

α-KG: alpha-ketoglutarate
AICAR: 5-aminoimidazole-4-carboxamide ribonucleotide
AMPK: AMP-activated protein kinase
ANOVA: analysis of variance
ATP: adenosine triphosphate
CASP3: caspase 3
DHODH: dihydroorotate dehydrogenase
DMSO: dimethyl sulfoxide
ELISA: enzyme-linked immunosorbent assay
FC: fold change
G6PD: glucose-6-phosphate dehydrogenase
GAPDH: glyceraldehyde 3-phosphate dehydrogenase
GPX4: glutathione peroxidase 4
GSH: glutathione
KD: knockdown
KEGG: Kyoto Encyclopedia of Genes and Genomes
LC-MS: liquid chromatography–mass spectrometry
MDA: malondialdehyde
MTBE: methyl tertiary butyl ether
MUFA: monounsaturated fatty acid
NAC: *N*-acetylcysteine amide
NCOA4: nuclear receptor coactivator 4
OA: oleic acid
PAGE: polyacrylamide gel electrophoresis
PARP1: poly(ADP-ribose) polymerase 1
PBS: phosphate-buffered saline
PDAC: pancreatic ductal adenocarcinoma
PI: propidium iodide
PPP: pentose phosphate pathway
PTGS2: prostaglandin-endoperoxide synthase 2
PUFA: polyunsaturated fatty acid
qPCR: quantitative real-time polymerase chain reaction
SCD: stearoyl-CoA desaturase
SCD5: stearoyl-CoA desaturase 5
SD: standard deviation
SLC25: solute carrier 25
SLC25A10: solute carrier family 25 member 10 (also known as DIC)
SLC25A11: solute carrier family 25 member 11 (also known as OGC)
SLC25A22: solute carrier family 25 member 22
STS: staurosporine
TCA: tricarboxylic acid
VIP: variable importance projection
