# Supplementary material for: SLC25A22 as a Key Mitochondrial Transporter Against Ferroptosis by Producing Glutathione and Monounsaturated Fatty Acids
Source: Antioxid Redox Signal. 2023 Jul 17;39(1-3):166–85. doi: 10.1089/ars.2022.0203 (PMC10620438; doi:10.1089/ars.2022.0203)
Supplement: Supplemental data [file Supp_FigS1.docx]

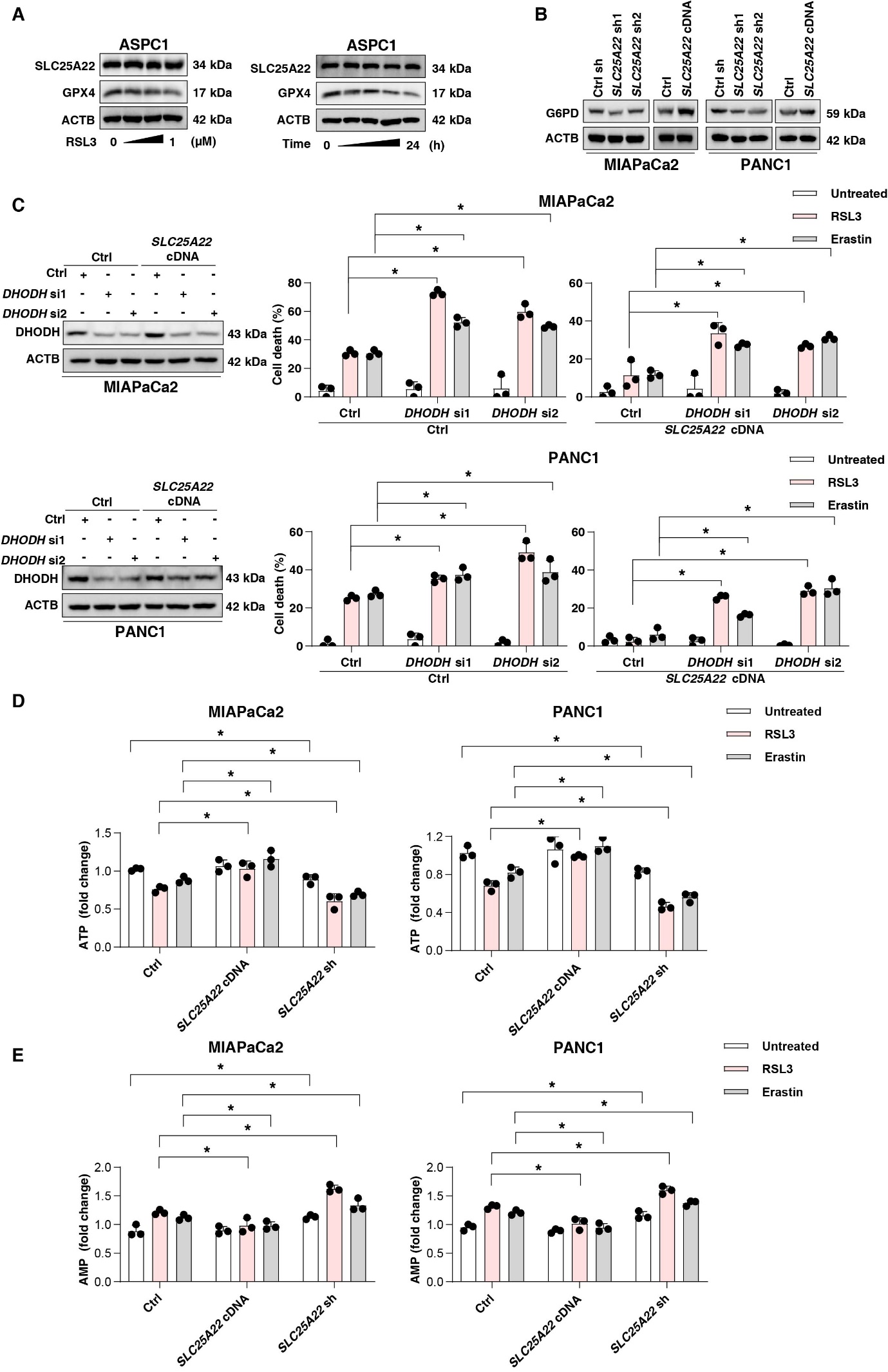


**Figure S1. SLC25A22 as a ferroptosis inhibitor in PDAC cells.**

(A) Analysis of SLC25A22 and GPX4 protein levels in indicated PDAC cells following treatment with RSL3 (0.25, 0.5, and 1 µM) for 24 h (left) or following treatment with RSL3 (1 µM) for different times (3, 6, 12, and 24 h; right). (B) Analysis of G6PD protein expression in indicated PDAC cells. (C) Analysis of DHODH protein expression in indicated PDAC cells (left). Cell viability of indicated PDAC cells following treatment with RSL3 (1 µM) or erastin (20 µM) for 24 h. (D-E) The indicated PDAC cells were treated with RSL3 (1 µM) or erastin (20 µM) for 24 h, and then the levels of ATP (D) and AMP (E) were measured. Data in C-E are presented as the mean ± SD of three technical replicates from one representative dataset of three independent experiments. Statistical significance was analyzed using two-way ANOVA with Dunnett's post-hoc test. **P* < 0.05.
